# Revitalizing the setting approach – supersettings for sustainable impact in community health promotion

**DOI:** 10.1186/s12966-014-0118-8

**Published:** 2014-09-14

**Authors:** Paul Bloch, Ulla Toft, Helene Christine Reinbach, Laura Tolnov Clausen, Bent Egberg Mikkelsen, Kjeld Poulsen, Bjarne Bruun Jensen

**Affiliations:** Steno Health Promotion Center, Steno Diabetes Center, Niels Steensens Vej 8, DK-2820 Gentofte, Denmark; Research Centre for Prevention and Health, Glostrup University Hospital, Nordre Ringvej 57, Building 84-85, DK-2600 Glostrup, Denmark; AAU-MENU, Meal Science & Public Health Nutrition, Department of Development and Planning, Aalborg University, A.C. Meyers Vænge 15, DK- 2450 Copenhagen, SV Denmark

**Keywords:** Setting approach, Health promotion, Local community, Integration, Participation, Empowerment, Sustainable development, Action research, Supersetting

## Abstract

**Background:**

The concept of health promotion rests on aspirations aiming at enabling people to increase control over and improve their health. Health promotion action is facilitated in settings such as schools, homes and work places. As a contribution to the promotion of healthy lifestyles, we have further developed the setting approach in an effort to harmonise it with contemporary realities (and complexities) of health promotion and public health action. The paper introduces a modified concept, the supersetting approach, which builds on the optimised use of diverse and valuable resources embedded in local community settings and on the strengths of social interaction and local ownership as drivers of change processes. Interventions based on a supersetting approach are first and foremost characterised by being integrated, but also participatory, empowering, context-sensitive and knowledge-based. Based on a presentation of “Health and Local Community”, a supersetting initiative addressing the prevention of lifestyle diseases in a Danish municipality, the paper discusses the potentials and challenges of supporting local community interventions using the supersetting approach.

**Discussion:**

The supersetting approach is a further development of the setting approach in which the significance of integrated and coordinated actions together with a participatory approach are emphasised and important principles are specified, all of which contribute to the attainment of synergistic effects and sustainable impact of supersetting initiatives. The supersetting approach is an ecological approach, which places the individual in a social, environmental and cultural context, and calls for a holistic perspective to change potentials and developmental processes with a starting point in the circumstances of people’s everyday life. The supersetting approach argues for optimised effectiveness of health promotion action through integrated efforts and long-lasting partnerships involving a diverse range of actors in public institutions, private enterprises, non-governmental organisations and civil society.

**Summary:**

The supersetting approach is a relevant and useful conceptual framework for developing intervention-based initiatives for sustainable impact in community health promotion. It strives to attain synergistic effects from activities that are carried out in multiple settings in a coordinated manner. The supersetting approach is based on ecological and whole-systems thinking, and stipulates important principles and values of integration, participation, empowerment, context and knowledge-based development.

## Introduction

Primary Health Care (PHC) has remained a priority on the global health agenda since the Alma Ata meeting [1]. The year 2008 thus celebrated 30 years of PHC policy with two major reports, “*The World Health Report 2008* - *Primary Health Care Now More Than Ever”* [2] and the report of the Commission on the Social Determinants of Health [3]. Both reaffirmed the relevance of PHC in terms of its vision and values in today’s world. However, the world has changed radically since 1978 and is now characterised by globalisation, urbanisation, rapid communication and an increasing gap between rich and poor. In the context of health and health care the world has seen a shift from major concerns about communicable diseases to chronic non-communicable diseases (NCDs) and thus from targeted single interventions to concerns about the environment, life style and behaviours of people; ideological changes as dictated by neoliberal economics and new public management, along with dominance of large monetary management institutions such as the International Monetary Fund (IMF) over the United Nations (UN) organizations; and a shift from medical professional monopoly on decisions and resource allocation to a much wider role for lay people [4]. Moreover, the poorest part of the world has experienced the emergence of resource requiring vertical health programmes such as national HIV/AIDS, TB and Malaria programmes and of wealthy philanthropic organizations with substantial influence on national public health agendas. This situation presents large challenges and demands serious rethinking about the PHC vision. The traditional model of PHC is no longer sustainable, requiring a revitalization that entails fundamental changes in how primary care is delivered and financed.

The Ottawa Charter for Health Promotion defines health promotion as the process of enabling people to increase control over, and to improve, their health [5]. It conceptualizes health promotion action as building healthy public policy; creating supportive environments; strengthening community actions; developing personal skills; reorienting health services (beyond its responsibility for providing clinical and curative services) and moving into the future (with caring, holism and ecology as central strategic elements). The Ottawa Charter further specifies that health promotion action “*has to be facilitated in schools, homes, work places and community settings”* because “*health is created and lived by people within the settings of their everyday life; where they learn, work, play and love”*. WHO defines a “setting” as a “*place or social context in which people engage in daily activities in which environmental, organizational and personal factors interact to affect health and well being*… *A setting is also where people actively use and shape the environment and thus create or solve problems relating to health”* [6]. Poland and colleagues [7] further argues that settings are both the medium and the product of human social interaction and thus more than simply locations in space-time. The setting approach thus emphasises the individual, social and structural dimensions of health promotion.

The WHO Global Strategy for Health for All by the year 2000 [8] together with the Ottawa Charter [5] provided important inspiration towards establishing the holistic and multifaceted approach embodied by Healthy Settings programmes, as well as towards the integration of health promotion and sustainable development [9]. The key principles of Healthy Settings include community participation, partnership, empowerment and equity. The Healthy Cities programme is probably the best-known example of a successful Healthy Settings programme. Initiated by WHO in 1986, Healthy Cities have spread rapidly across Europe and other parts of the world [10]. The successes of settings-based approaches have been validated through internal and external evaluation and experiences [9]. On this basis WHO argues that Healthy Settings remains a useful, dynamic method to integrate risk factors and address disease prevention aiming to improve overall quality of life.

Factors influencing our health and quality of life are numerous and have been categorised by Whitehead and Dahlgren [11] as 1) biological, physical and constitutional such as age and sex, 2) individual and lifestyle related, 3) social and network related, 4) related to living and working conditions, and 5) political, socioeconomic, cultural and environmental. In a world where most high and middle income countries are struggling with the prevention and control of NCDs and where low-income countries will barely overcome the challenges of infectious diseases before NCDs are emerging as development and prosperity rises (giving rise to the so-called double burden of disease), the need to rethink disease prevention and health promotion strategies is bigger than ever. NCDs are characterised by their chronic nature and this puts an excessive and long-term burden on national health systems and budgets across the spectrum of low, middle and high income countries [12]. Sustainable successes in the prevention of NCDs are mainly involving structural and regulatory interventions such as increased taxation (on tobacco, alcohol etc.) and restrictions in the amounts of additives to food products (e.g. salt, trans-fats etc.) [13]. Such interventions are often effective in reducing disease incidence but may suffer from being authoritarian and in conflict with consumer interests. It has also been argued that taxation affects the poorest hardest and may thus be socially inequitable [14]. It may therefore be more socially equitable and politically viable to strive towards affecting people’s attitudes, motivations and practices in efforts to influence healthy living. Our attitudes, motivations and practices are formed by influences from a variety of people (e.g. parents, friends, employers, colleagues, teachers and media professionals) and conveyed in a variety of settings (e.g. schools, homes, neighbourhoods, work places, clubs, social networks and media). Our lifestyles, life qualities and health are thus not isolated and independent phenomenon’s, which can be adjusted and modified based exclusively on personal desires and needs. Our lifestyles, life qualities and health are also products of our life circumstances, social interactions and attention paid to societal discourses and diverse natures of influences affecting our senses.

In concordance with the increased or renewed recognition of the importance of the principles of PHC, health promotion action, and the social determinants of health, we have, as a contribution to the promotion of healthy lifestyles, further developed the setting approach in an effort to harmonise it with contemporary realities (and complexities) of health promotion and public health action. The paper introduces a modified concept, the supersetting approach, building on the optimised use of diverse and valuable resources embedded in local community settings and on the strengths of social interaction and local ownership as drivers of change processes. Based on a presentation of an ongoing initiative addressing the prevention of diabetes and other lifestyle diseases in a Danish municipality the paper discusses the benefits and challenges of supporting local community partnerships using the supersetting approach.

### The supersetting approach

#### The conceptual framework

Health should be promoted within the settings of people’s everyday life because this is where people engage in daily activities and this is where environmental, organizational and personal factors interact to affect health and well being [5]. This important recognition from the early days of PHC is the foundation upon which the setting approach rests. It is also the foundation upon which the supersetting approach rests. The supersetting approach is an intervention strategy whereby coordinated activities targeting a common overall goal such as improved health in a population group are carried out in a variety of different settings and involving a variety of different stakeholders within a local community. The supersetting approach is more than a multi-setting approach. The supersetting approach strives to attain synergistic effects from operations that are carried out in multiple settings either simultaneously or phased but always in a coordinated manner. Furthermore, the supersetting approach cannot be implemented as a top-down model by e.g. researchers or city planners, but demands the active participation of local stakeholders. This has the advantage of bringing several community resources into play while preventing antagonistic action by opposing forces. As an example, efforts to affect the smoking behaviour among adolescents may benefit from coordinated efforts at schools, social media, sports clubs and supermarkets, but also at the work places of their parents. Without such multi-setting interventions the smoking patterns or attitudes of the parents may be counterproductive to the efforts in other settings to prevent smoking among adolescents.

Interventions based on a supersetting approach are first and foremost characterised by being integrated, but also participatory, empowering, context-sensitive and knowledge-based.

#### Integration

Effective and sustainable development is supported by an integrated approach to action planning, implementation, monitoring and evaluation. Integration refers to the *coordination* and, if possible, co-implementation of activities that share features in relation to applied methods, targeted populations, timing, expected outcomes etc. It also refers to the *assimilation* of values, approaches, procedures and standards in established structures and cultures of organisations in the local community and larger society. Finally, integration refers to the *cooperation* of stakeholders with diverse backgrounds and professions but acknowledging the interrelatedness and inter-sectoral nature of challenges facing society in the 21st century. The purpose of integration is thus to contribute lasting organisational value to stakeholders while achieving synergy in their achievements. The process of integrating health promotion initiatives, which often involves a variety of professions, sectors and disciplines, is obviously difficult but may be optimised by the early establishment of long-term cross-cutting coordination groups for stakeholder representatives. Once common levels of understanding of each other’s values, norms and aspirations are reached, these groups may enjoy flexibility in trying and testing bold ideas that would be difficult within the framework of individual mother-organisations. Sustainable integration of health promotion action cannot be forced but depends on the establishment of such mutual respect, trust and understanding of the needs and benefits of working together, by giving and taking, in order to reach a common overall goal. The Adelaide Statement on Health in All Policies proposes a new form of governance where there is “*joined-up leadership within governments, across sectors and between levels of government*“ in efforts to improve health outcomes and advance human development, sustainability and equity [15]*.* In recognition of this notion, the supersetting approach argues for optimised effectiveness of health promotion action by integrating efforts in intersectoral partnerships involving a diversity of relevant sectors such as health, environment, education, politics and finance.

#### Participation

Attitudinal and behavioural change of ordinary people not only requires knowledge and insight to alternative ways of thinking and acting but also psychological adaptation to norms and recommendations for better ways of living. The process of acquiring new knowledge and adapting psychologically to new recommendations is fuelled by motivation, and motivation is stimulated by active involvement and participation, which create a feeling of ownership of change processes. The supersetting approach thus argues for a high degree of participation of beneficiaries (target groups) in developing, implementing, monitoring and evaluating health promotion initiatives to increase the likelihood of achieving sustainable attitudinal and behavioural change. Moreover, the supersetting approach argues for *inclusiveness* in terms of informing, involving, engaging and partnering with as many community stakeholders (i.e. institutions, organisations, associations and companies within the private sector, the public sector, political systems, academia, civil society and the media) as possible. This is because of the wide availability, in all communities, of resources that are relevant for health promotion action. Resources can be material or financial, or they can relate to the dedication of time, expertise or creative thinking by ordinary citizens or professionals alike.

#### Empowerment

The supersetting approach emphasises a sustainable development rationale embedded in empowerment principles. The supersetting approach argues for promoting attitudinal and behavioural change by establishing and facilitating respectful dialogue with people, and subsequently making opportunities and support available to them, on how to acquire relevant knowledge, skills and experience in a particular subject. Empowerment is being properly promoted when people succeed in optimising their ability to define and argue personal attitudes, values and goals, and to act and take responsibility thereafter in a proper balance between personal integrity, social norms and societal rules and regulations. This is obviously time-consuming. People may be (and most often are) aware of their own unhealthy lifestyle, and of personal measures that *should* be adopted to improve it, but may lack the motivation and/or competences to take action. The deeper causes behind de-motivation and in-competences are to be found in the social determinants of people and their everyday pressures, and these are not modifiable over night. The supersetting approach recognises that people’s attitudes and behaviours are deeply rooted in people’s social contexts and systems and that these take a very long time to modify. In recognition of these difficulties, proper supersetting initiatives are bringing people to the centre of long-term social development processes based on respectful dialogue, competence building opportunities and motivating action.

#### Context

The political, social, economic, environmental and cultural contexts in the local community or in society at large are important attributes affecting attitudinal and behavioural change potentials and processes of ordinary people. Contextual factors can either be conducive or disruptive for health promotion efforts and should therefore be understood and, if possible, accounted for in the planning and implementation of supersetting initiatives. These factors may relate to structural (e.g. regulatory, legislative or financial) circumstances affecting the opportunities of stakeholders such as schools or sports clubs to engage in health promotion actions. In most instances it is possible for health promotion initiatives to understand and document these contextual factors but not to influence them. However, when attempts to modify structural factors are successful, this may effectively stimulate behavioural change. Contextual factors may also relate to the circumstances of everyday life as perceived by beneficiaries (people or populations groups) targeted by health promotion activities. In this case, context may comprise very local level barriers and opportunities at the level of the household, classroom, or local community. Examples are the presence/absence of active citizens dedicated to social mobilization for healthy living, the presence/absence of policies and strategies for healthy eating and physical activity in schools and day-care institutions, and the presence/absence of physical spaces and environments in the local community, which are conducive for healthy living. Such contextual factors should be understood and addressed through direct interaction and dialogue with beneficiaries, by identifying what is relevant, interesting and realistic in their view, and by jointly planning and implementing agreed upon activities.

#### Knowledge-based interventions

The supersetting approach is knowledge-based. It applies and produces scientific knowledge of highest standard within the framework of any respected research tradition and scientific discipline of relevance to the subject, e.g. natural, medical, social and humanistic sciences. State-of-the-art knowledge and experience is extracted from the scientific literature and used to inform the design of interventions. Moreover, scientific knowledge is produced by monitoring and studying the qualities of change processes and by determining the effects of interventions. Complex interventions in local community settings do not follow simple linear cause-effect relationship. Evaluating such interventions is therefore a challenging process for which a theory-driven evaluation method such as “realist evaluation” is a useful alternative to randomized controlled trials. Action research (and related participatory research methodologies) is a process of inquiry of particular importance to the supersetting approach because it builds on the active involvement of target groups (beneficiaries) in designing interventions and in iteratively evaluating and adjusting them during the course of a supersetting initiative. Applying action research thus complements other supersetting principles of participation, empowerment and action competence. The intention is to make disciplines meet and interact, and, in crossing their conventional boundaries, generate new and innovative approaches, interventions and solutions as well as broadening the scope and nature of findings from studying their processes and outcomes. Returning to the above example, efforts to affect the smoking behaviour among adolescents may include pedagogical intervention, health education, (mass and social) media intervention, social mobilisation, structural and regulatory intervention etc. Each of these interventions may benefit from epidemiological and register-based knowledge about the magnitude of smoking among adolescents, from detailed understanding of barriers and opportunities to cessation of smoking through qualitative in-depth interviews or focus group discussions, from active engagement of adolescents in defining and implementing solutions through action research, and from a broader understanding of smoking-related knowledge, attitudes and practices of adolescents before, during and after interventions through quantitative questionnaire surveys. The supersetting approach thus argues for interdisciplinary initiatives and theory-driven evaluation methodology in order to optimise interventions and enrich the findings.

**Figure 1:**
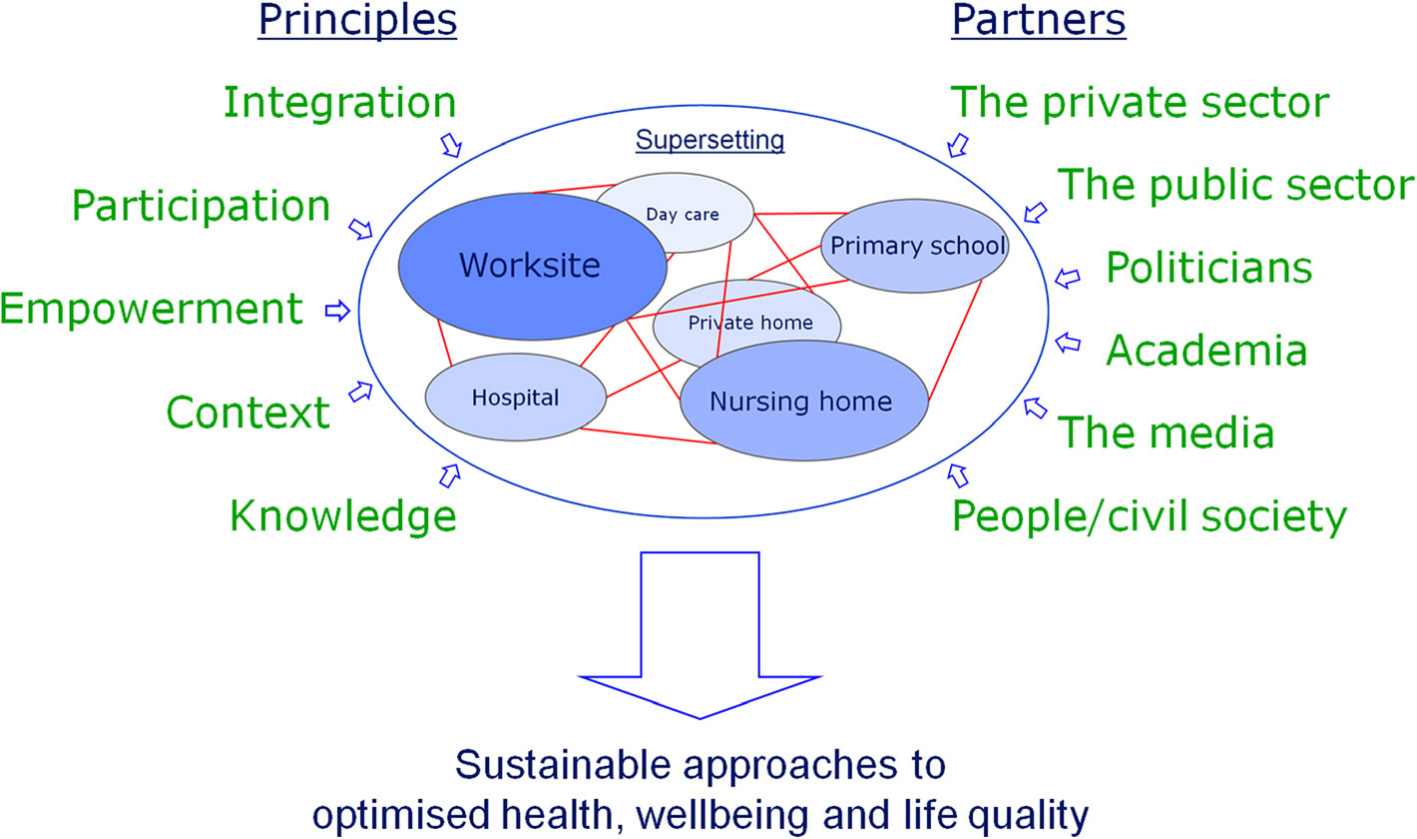
**The supersetting approach: Applying a set of principles (listed on the left hand side) and involving relevant partners (listed on the right hand side) within the supersetting (centred circle) as the basis for developing sustainable approaches to optimised health, wellbeing and life quality.** The supersetting is represented as multiple settings within a local community (the outer ring). Activities within individual settings are coordinated and integrated (symbolised by the lines) with activities in other settings as the basis for achieving synergistic effects.

When applying all of the above-mentioned principles and involving all relevant stakeholders, the supersetting approach provides a useful conceptual framework for sustainable health promotion action (Figure 1). Its specific elements are not new but so is the way they are structured. The supersetting approach thus builds on the best features of the setting-approach and of participatory and ecological whole-systems approaches. However, as a supplement to the setting approach, the supersetting approach insists on bringing very different community stakeholders (professionals as well as ordinary citizens) together for jointly developing, planning, organizing and implementing integrated health promoting actions across settings and across the wide spectrum of political, economic, social, professional and environmental interests; and as a supplement to the ecological whole-systems approaches, the supersetting approach insist on empowering community stakeholders through structured participatory development and implementation processes respecting the challenges of every-day life circumstances and fostering local ownership, motivation, responsibility and competences to act for a common cause. Sustainable health impact originates from the combination of these important principles of the supersetting approach.

#### The process of organising a supersetting initiative

A supersetting initiative is broadly owned by all of its stakeholders. Everyone must have a say in terms of influencing content and direction of the initiative. This necessitates the formation of a comprehensive organisation structure for dialogue, decision-making, coordination and action between ordinary citizens (beneficiaries), civil society organisations, public authorities and their institutions, private sector corporations, and researchers.

There are two main pathways through which a supersetting initiative can evolve. In a *top-down pathway*, a central core of professional organisations, institutions and/or corporations get together in response to a certain challenge, demand or idea, and establishes a joint communication and coordination forum (e.g. a steering committee) in which major strategic decisions are taken. A number of sub-structures and work groups are subsequently established. These have different roles and functions related to planning, implementation, monitoring, evaluation and research, and are therefore represented by different stakeholders, some of which are ordinary citizens. Over time, as the initiative matures and more stakeholders get onboard, the aim (and challenge) is to maintain a certain degree of overall formal coordination of activities while securing a high degree of communication between the organisational units as well as a high degree of autonomy for all units to take meaningful decisions and to be able to act on them. In a *bottom-up pathway*, the need for change emerges from interaction and dialogue among beneficiaries such as parents, elders, youth or ordinary citizens as a whole. A more or less informal community forum is established to transform ideas into a coherent strategy and plan, and to mobilise internal and external resources for their implementation. The aim (and challenge) is to operate at a grass root level and reduce outside dominance while attracting sufficient attention and interest among public and/or private authorities and funders to be able to implement what is planned. It is difficult to imagine an effective and sustainable supersetting initiative without elements of both top-down and bottom-up characteristics. Commitment from public and private sector authorities is required because they grant permission to involve their organisations and institutions such as schools, kindergartens, museums and libraries in an initiative, and they represent more or less permanent organisational structures that are resistant to fluctuations in personal motivations and commitments of involved citizens or professionals. Similarly, commitment from citizens and other target groups is required because these are the people who can best, from both an ethical and a practical perspective, define the needs and visions for their community, secure relevance and legitimacy of an initiative, and foster local ownership, social responsibility and sustained motivation to take action. The deeper the gap between the interests of societal authorities and its citizens, between formal organisations and informal social movements, the more difficult it becomes to manage and implement a supersetting initiative. No matter which top-down and bottom-up balance is applied, a proper supersetting initiative must support bold ideas and risk-taking, acknowledge the diverse competences and functions of professional stakeholders, and fully respect and involve its beneficiaries, the prime target group of citizens, in order to succeed and bring about sustainable impact in health promotion.

### The case: a Danish supersetting initiative

To illustrate the prospects and challenges of the supersetting approach, this section briefly presents an ongoing supersetting initiative with emphasis on how it was formed and how it complies with the values and principles of the supersetting approach. The initiative is called “Health and Local Community” and is carried out in three local communities in the Danish municipality of Bornholm, an island with a land mass of 588 square kilometres and a population size of approximately 42.000 inhabitants. Most health and social indicators of the population are below average for the country. “Health and Local Community” is a research and development initiative aiming at influencing the lifestyle habits of families with small children aged 3–8 years with emphasis on mobilising community resources, strengthening social networks, and promoting healthier food choices and more physical movement. Apart from the main target group of families with small children, the initiative involves several local stakeholders and settings, most notably professionals within primary schools, after-school centres, childcare centres, supermarkets, media and a number of civil society organisations and resource persons with expertise in nutrition, cooking, recreation and physical movement. The initiative also involves three Danish research institutions with various levels of expertise in public health, epidemiology, social science and education. “Health and Local Community” receives most of its funding from a private Danish charity foundation, the Nordea-fonden.

“Health and Local Community” has been designed to comply with the values and principles of a supersetting initiative. This is illustrated as follows:

- *Integration*. The initiative implements coordinated and integrated interventions in local primary schools/childcare centres, supermarkets and media. This is done in a formal partnership between well-established organisations, institutions and private enterprises in the local community. The partnership includes three departments within local government (i.e. health/social services, education/day-care and leisure-time/prevention), a local NGO engaged in community development (and hosting the local coordinator of the initiative), three supermarket chains with outlets/shops in the targeted communities, and the local TV station. Furthermore, the initiative has established local action groups for professionals (e.g. school teachers, shop owners, fitness instructors etc.) and citizens working and/or living in the targeted communities. These local action groups serve as coordination and mobilisation forums for community arrangements that are identified, planned and implemented through voluntary engagement. The broad representation of participants in the local action groups allow for the implementation of activities that are truly community-based and community-involving rather than setting-specific. The formation of local action groups is therefore an important operational step, which will foster synergistic actions across settings and optimise their local relevance, integration and sustainability in line with the principles of the supersetting approach.
- *Participation*. The development of interventions is based on the use of participatory methods involving local stakeholders, most importantly the prime target group of families with small children. Participation takes place at several levels and in different locations and situations. Although variation occurs, the tendency is that 1) evaluations and strategic planning are carried out at joint annual meetings involving high-level decision-makers, managers and in-charges of formalised partners, 2) six-months thematic planning involves managers, in-charges and professional employees of formalised partners, 3) activity planning and implementation involves local professionals, resource persons and citizens on a case-to-case basis. Moreover, participation either occurs around setting-specific arrangements implemented in places such as schools, kindergartens or supermarkets (and involving children, parents, grandparents, customers, professionals etc.) or around truly cross-cutting community arrangements organised by participants of the local actions groups. The researchers support these local processes by presenting ideas for inspiration of local stakeholders, by contributing scientific knowledge about relevant issues such as nutrition and physical movement, and by facilitating meetings and training courses according to locally defined needs.
- *Empowerment*. The initiative supports empowerment processes through social learning and action-competence building [16]. The most important target group for empowerment processes in the initiative are children. By use of participatory learning methods such as future workshops [17] children are engaged in processes of identifying and solving problems in their local environment. We mainly use visionary representations in the form of photos, drawings, collages and physical models to stimulate reflection and expression of opportunities, visions and ideas that would make the local environment such as class room, canteen or outdoor physical space more attractive, interesting, fun and healthy to use. These are presented to wider audiences of relatives and professionals, and key priorities emerging from the processes are brought forward by the initiative and turned into concrete projects for implementation in and by the local community. Children thereby experience a connection between their own visions and expressions for a better physical and social environment, and subsequent responses and actions by adults. Whereas this builds action competence in children, the initiative also provides capacity and training to professionals to sustain these processes in iterative cycles of participatory engagement of children and adults, and joint evaluations and adjustment of actions.
- *Context*. The initiative benefits from its wide network of local stakeholders, including its relationship to local mass media (TV, radio and newspapers), to monitor contextual factors that may influence the realisation of planned activities. These factors relate to community and societal developments and influences such as changes in local government priorities, structural adjustment plans, sector-specific budget changes, related development initiatives, mass media agendas, and weather conditions as well as very local developments and influences such as institutional planning cycles, staff changes, motivations of citizens, and local community events. The initiative constantly re-plans and re-organises according to changing circumstances, and actively uses contextual information to avoid clashes of interests and to engage in local events such as public meetings, debates and other arrangements.

**Figure 2:**
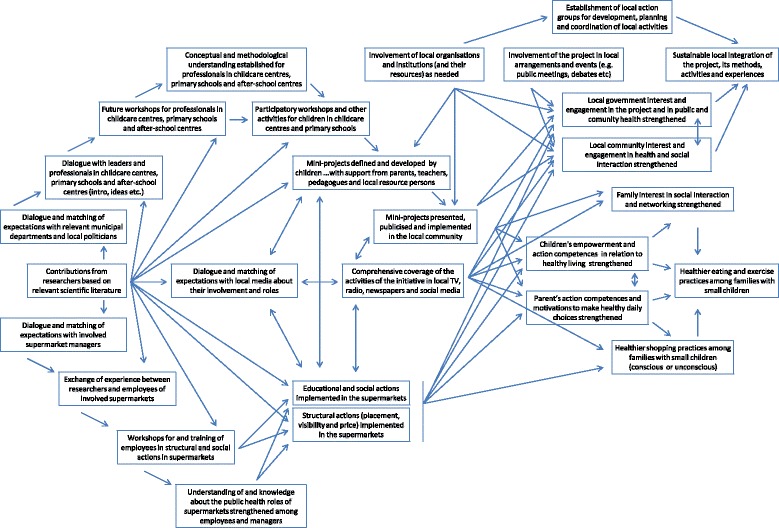
**The conceptual framework of the Danish supersetting initiative “Health and Local Community” carried out in the municipality of Bornholm.** Arrows represent expected cause-effect relationships. Actions are shown on the left hand-side of the figure, outputs are shown in the middle and outcomes and effects are shown on the right hand-side.

The conceptual framework of “Health and Local Community” is outlined in Figure 2. A main feature of the conceptual framework is its high level of complexity, which is a result of the holistic nature of the initiative and the wide involvement of local stakeholders. Three main pathways of actions and outputs are apparent in the figure. One of these pathways illustrates actions and outputs within schools and child care centres. Here efforts seek to involve children, parents, grandparents and professionals in defining and implementing solutions that promote healthy living by reconstructing the physical and social space within the institutions and, more widely, within the local community. Another pathway illustrates actions and outputs within supermarkets. Here efforts seek to involve shop owners and staff as well as customers (mainly families with small children) in defining and implementing ways of restructuring the shops for the purpose of promoting sale of healthy commodities (such as whole grain products and vegetables) and discouraging sale of unhealthy commodities (such a sweets and soft drinks). The final pathway illustrates actions and outputs related to the involvement of media. Here efforts seeks to engage local radio, TV and newspapers to report from, and actively participate in, all kinds of activities organised by “Health and Local Community” including sports events, nature walks, cooking workshops, fishing trips etc. It is noted from the conceptual framework that the three pathways are connected by arrows. This indicates that activities are organised across the different settings. As an example, school children are invited to local supermarkets to prepare their own packed lunches based on a wealth of healthy food products made available by the shop owners. This provides opportunities for school teachers to teach about nutrition and healthy eating in an alternative and very conducive environment. It also provides inspiration to parents who acquire ideas for preparing more interesting and healthy lunches to their children. Supermarkets thus functions as new social learning platforms for the children outside the traditional classroom. As another example, childcare centres and nature guides organise nature walks for children and parents with a focus on edible resources within the local environment; this is followed by outdoor cooking using nature’s ingredients and, subsequently, treasure hunts in supermarkets for equivalent food products. Local media closely cover these activities and debate them with lay people and experts in thematic programmes on healthy living. It is also noted from the conceptual framework that expected outcomes and effects of all these actions tend to merge and are not associated with one particular pathway. This illustrates a high degree of coordination and planning for synergistic effects and common goals, which is an inherent element of a supersetting initiative.

**Figure 3:**
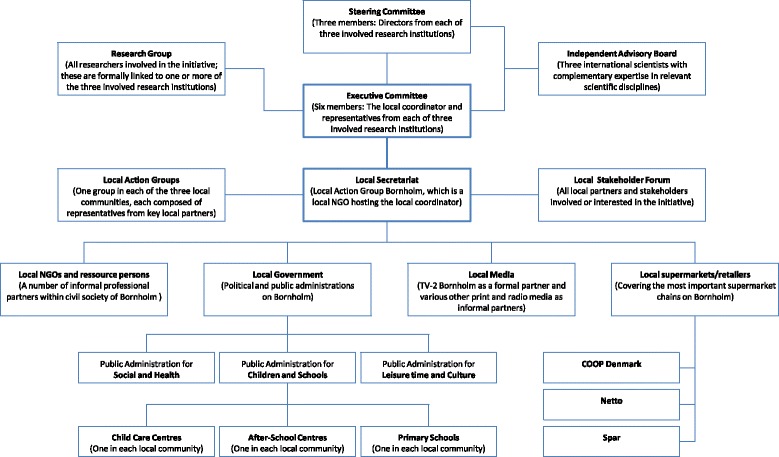
**The organisation structure of the Danish supersetting initiative “Health and Local Community” carried out in the municipality of Bornholm.**

The organisational structure of “Health and Local Community” is outlined in Figure 3. The initiative is organised as a formalised partnership with key stakeholders shown in the figure. Informal partners in civil society (i.e. NGO’s and independent professional resource persons) are not listed in the organisational structure. The various boxes represent partners, settings, coordination groups and an independent advisory committee. An executive committee functions as the engine of the initiative with responsibility for day-to-day planning and coordination. This committee is the only organisational unit, which includes members from both arms of the initiative, namely the development arm and the research arm. It is through the executive committee that development and research agendas are synchronised and more widely communicated within the organisation structure.

During the first one and a half years of the intervention phase numerous activities have been implemented in all of the involved community settings. Jointly identifying and organising these activities with local stakeholders required massive dialogue and matching of expectations. At the beginning, therefore, limited attention was paid to securing synergistic actions and coordination across settings and professions. This is now changing and the initiative is currently entering a phase of transition. The initiative has become well-known across the island. Trust has been established between partners. It has been widely recognised that stakeholders have many diverse agendas and motivations for joining the initiative, and that no single stakeholder can have all of its ambitions fulfilled. Public and private authorities have thus acknowledged their positions as partners rather than owners of the initiative. Professionals in schools, childcare centres and supermarkets have realised that the initiative not only *consumes* staff time but also *contributes* resources in the form of creative ideas and tangible assistance from local stakeholders, external resource persons and researchers alike. The prime target group of children, as well as most of their parents and grandparents, are very attentive towards the initiative and engage themselves in various ways, e.g. by participating in arrangements, defining and shaping arrangements, producing accessories for arrangements, evaluating arrangements, communicating arrangements etc.

## Discussion

This paper introduces a new concept, the supersetting approach, in an effort to further develop the concept of the setting approach. Revitalizing the setting approach is justified by the dramatic changes in health governance that has taken place since the dawn of Primary Health Care and the Ottawa Charter for Health Promotion around 30 years ago. Health governance in the 21st century is strongly influenced by globalisation, which is characterised by a growth in the number and degree of influence of non-state actors (including civil society groups, global social movements, private companies, consultancy firms, think tanks and religious movements). New combinations of both state and non-state actors are rapidly forming in a myriad of forms such as partnerships, alliances, coalitions, networks and joint ventures. Globalisation increases connectedness and interaction and thus provides major opportunities for improvement in public health. However, globalisation has also been shown to worsen poverty, marginalisation and health inequity [18]. This has been referred to as “global public bads” arising largely from ineffective health governance [19]. “Health for all” by the year 2000 was not achieved, and the Millennium Development Goals (MDGs) for 2015 will not be met in most low-income countries due to insufficient political prioritisation of health, structural adjustment policies, poor governance, population growth, inadequate health systems, and scarce research and assessment on primary health care [20]. Kickbusch [21] argues that we are at a turning point in health policy: The nature of 21st century health, changes in society and technology call for a radical change of mind set and a reorganisation of how we govern health. Health promotion is challenged to link three big debates to the health agenda, namely wellbeing, sustainability and social investment. The setting approach is an integral element of health promotion and provides important values, principles and learning opportunities for meeting the new challenges for health governance and health promotion in the 21st century.

The supersetting approach is not representing a radical shift in thinking about the potentials and powers of the setting approach. The supersetting approach builds on the massive amount of work done by others since the days of the Ottawa Charter. It is based on important lessons learnt from ecological and whole-systems initiatives, and brings key values, principles and experiences from the past together in a new way. Furthermore, it emphasizes the need for involving all stakeholders (including the target groups) in developing the approach. In a detailed review of the diversity of approaches applied in settings based health promotion initiatives, Whitelaw et al. [22] outlines five types of models, namely the “passive” model, where the problem and solution rest within the behaviour and actions of individuals; the “active” model, where the problem lies within the behaviour of individuals, and part of the solution lies in the setting; the “vehicle” model, where the problem lies within the setting, and the solution in learning from individually based projects; the “organic” model, where the problem lies within the setting, and the solution in the actions of individuals; the “comprehensive/structural” model, where the problem and solution lie in the setting. These models represent different degrees of engagement of the setting, from merely using the setting to access individuals at the one end to actively engaging and influencing the setting and its resources at the other. The supersetting approach mainly builds on the “organic” and “comprehensive/structural” models and may be viewed as a development, a modified version 2.0, of the setting approach in which the significance of integrated and coordinated actions is emphasised and important principles are specified, all of which contribute to the attainment of synergistic effects and sustainable impact on health and wellbeing. The supersetting approach is thus more than a multi-setting approach. The supersetting approach is an ecological approach, which places the individual in a social, environmental and cultural context, and calls for a holistic and participatory perspective to change potentials and developmental processes with a starting point in the circumstances of everyday life. This rationale is strongly inspired by Bronfenbrenner’s [23] ecological systems theory and by Whitehead and Dahlgren’s [11] model on the determinants of health. Central to these theories is the holistic perspective on human beings and the interrelationship and connectedness between human beings and the biological, social, environmental, cultural, economic and political factors affecting their development and health. Based on the observation that a problem manifest in one setting may have its roots in other settings, Dooris [24] presented a rationale for connectedness whereby synergistic effects between settings may be obtained when effective action is taken in multiple settings. Practical examples of connectedness of the setting approach “outwards” (i.e. through coordination and joining-up of setting initiatives), “upwards” (i.e. through setting initiatives acting on the determinants of health) and “beyond health” (i.e. through collaboration between health and non-health setting initiatives) are available in the scientific literature but, Dooris [25] argues, the setting approach will only realise its full potential on sustainable health and wellbeing of people if it adopts a truly ecological approach, building bridges between silos of different programmes and networks, and reconfigures itself for the globalised 21st century.

Specific values and principles of the supersetting approach are associated with the concepts of integration, participation, empowerment, context and knowledge. These are discussed below.

Integration is a prime determinant of effectiveness and sustainability of interventions in community health promotion initiatives and therefore a fundamental feature of a supersetting initiative. In recent years there has been substantial international attention to the needs, prospects and potentials of strengthening intersectoral governance for health in all policies [26,27]. The Adelaide Statement on Health in All Policies outlines the need for a new social contract between all sectors to advance human development, sustainability and equity, as well as to improve health outcomes [15]. The statement emphasises that government objectives are best achieved when all sectors include health and well-being as a key component of policy-development. Moreover, Poland and Dooris [28] observes that there is a growing realization that the relationship between humans and the wider environment is crucially important, and, catalyzed particularly by concerns about climate change, that public health and the health of the planet are closely interrelated. On this basis the authors propose six principles for progressive practice as a means of grounding a healthy and sustainable setting approach: 1) Adopt an ecological “whole system” perspective, 2) meet with people where they are and understand their lived experience, 3) root actions and practice in the place, 4) deepen the social analysis, 5) apply asset-based and appreciative inquiry approaches, and 6) build resilience instead of efficiency. The health promotion and healthy settings literature suggests that health promotion in general [29] and healthy settings in particular [30] must embrace complexity and appreciate wholeness and interconnectedness. Dooris [31] further noted that effective action to address complex 21st century public health issues requires holistic system-based responses. In a white paper on present and future challenges for health promotion and sustainable development, Kickbusch [32] observes a need to move from a “silo” to a “systems” approach and argues that the purpose of governance in the 21st century should be healthy and sustainable development. Whilst the relationship of systems theory to complexity has been questioned [29], it has also been argued that systems thinking allows one to “do justice to the complexity of health” [33]. Health promotion is a social or “open” system, which interacts with its environment and responds to changes within and outside the system. Although it is difficult to gain insight into such social systems because of their complex and ever changing nature, important learning may be facilitated by addressing their structure, meaning and power relations.

Participation is a prime determinant of sustainable community action in community health promotion initiatives and therefore a fundamental feature of a supersetting initiative. Bracht & Tsouros [34] summarise on the benefits and difficulties of engaging citizens in community development activities and present a set of actions, which may enable people to become better organised, mobilise community resources and energies, and achieve a more effective participation in official decision-making mechanisms. Bracht & Rice [35] provide an account of community involvement strategies applied in projects around the world and present a detailed five-stage model including references to tools and materials for how to mobilise communities. The authors conclude that key factors contributing to successful citizen mobilisation include: 1) early commitment of project leaders to partnership and community development approaches, 2) clearly defined decision-making authority of citizen groups, 3) early establishment of a volunteer management and training program, and 4) timely use of conflict resolution strategies. In a comprehensive review of studies on participation in development, Mansuri & Rao [36] observed that community engagement alone has little impact on health outcomes but can substantially amplify the impact of investments in other health inputs. Interestingly, they use the example of the formation of community health groups, which, they observe, have virtually no effect on any health-related outcome when done in isolation but is effective when combined with other inputs such as trained health personnel or the upgrading of health facilities. Local action groups formed within the framework of “Health and Local Community” may serve a similar complementary purpose. They involve a very diverse range of community stakeholders and appear to foster synergistic actions across settings and to optimise the local relevance, integration and sustainability of community interventions. We argue that without a strong element of participation and social interaction among community stakeholders the diverse range of individual stakeholder interests may challenge the cohesion and resilience of any health promotion initiative. Mansuri & Rao [36], however, find little evidence that induced participation builds long-lasting cohesion, even at community level. They note that because similar types of people tend to form groups with one another, projects rarely promote cross-group cohesion, and may actually reinforce existing divisions. Rifkin [37] observes that community participation in health programmes has rarely met the expectations of health planners and professionals because community participation has been considered a magic bullet to solve problems rooted both in health and political power. For this reason, Rifkin argues, it is necessary to use a different paradigm which views community health promotion as an iterative learning process allowing for a more diverse approach to be taken.

Empowerment is a prime determinant of individual and social action in community health promotion initiatives and therefore a fundamental feature of a supersetting initiative. Community participation and empowerment are also core principles underpinning the Healthy Cities movement and an integral part of long-term strategic development [38,39]. Evaluations of the WHO European Healthy Cities Network have thus demonstrated that almost all of the involved cities had mechanisms for community representatives to participate in decision-making; and more than two-thirds of the cities had initiatives explicitly aimed at empowering local people. Recent publications affirm that community participation and empowerment have important benefits through increasing democracy, mobilizing resources and energy, developing holistic approaches, reducing inequity, achieving better decisions and more effective services, and ensuring ownership and sustainability of programs [40-42]. Nevertheless, Mansuri & Rao [36] observe that donor-driven participatory projects, most often conditioned by bureaucratic imperatives, declare that clear, measurable, and usually wildly optimistic outcomes will be delivered within a specified timeframe. They argue that repairing civil society and political failure are very difficult tasks that require a fundamentally different approach to development, namely one that is flexible, long term, self-critical, and strongly infused with the spirit of learning by doing.

Context may have dramatic effects on the development and change potentials and processes of people, projects and organisations alike, both positively and negatively. Context is thus a prime determinant of the efficiency and outcomes of community health promotion initiatives and therefore a fundamental feature of a supersetting initiative. Informed by ecological models of health it has been argued that the settings approach reflects an understanding of the importance of context in understanding both the socio-spatial distribution of health and the implementation (and effectiveness) of health and social care interventions [28]. Mansuri & Rao [36] observes that context, both local and national, is extremely important for the outcomes of participatory development initiatives. They argue that outcomes from community interventions are strongly influenced by a range of contextual factors (such as local inequality, history, geography, the nature of social interactions, networks and political systems) and that the variability of these factors is sometimes so large, and their effects so unpredictable, that projects that function well usually do so because they have strong built-in systems of learning and great sensitivity and adaptability to variations in context.

Knowledge based on scientific inquiry about the qualities of change processes and effects of interventions advances learning and understanding about what works and what does not work, for whom, when, where, why and how. Knowledge based on scientific inquiry is thus a prime determinant of learning from community health promotion initiatives and therefore a fundamental feature of a supersetting initiative. Generating such knowledge is, however, a major challenge. Supersetting initiatives are, and should be, based on complex interventions that are often interrelated, carried out in different settings, involving different professionals and, at times, targeting different population groups. Cause-effect relationships are often non-linear and only indirectly related to the overall objective of the initiative. It is the combined influence of numerous more or less distinct interventions that are believed to gradually induce change in people’s knowledge, perceptions, attitudes and behaviours. This is considered conducive, if not imperative, for promoting sustainable change in community-based health promotion initiatives but it is also a weakness in the effort to understand the influences and effects of *individual* interventions. As a supersetting initiative based on complex interventions “Health and Local Community” is evaluated by use of a theory-driven evaluation approach. The concept of theory-driven evaluation emerged during the 1980s in response to inadequacy of quasi-experimental research and evaluation designs, and to the need for proper approaches to evaluate the processes and effects of complex interventions [43]. Theory-driven evaluation includes different approaches such as “theory of change” [44,45] and “realist evaluation” [46], which are rooted in the notion that any specific intervention or planned action in a programme is associated with a theoretical reflection or hypothesis about a cause-effect relationship. A full programme theory comprise a comprehensive set of such interventions, each of which can be described and evaluated individually from the perspective of its own inherent logic about “what works, how, under which conditions and for whom”. Although it has been questioned whether theory-driven evaluation lives up to its promise [47,48], we have found it very useful to apply the realist evaluation approach to dissect and evaluate complex interventions in “Health and Local Community”.

Setting up the “Health and Local Community” initiative has been a challenge. The initiative was defined by research institutions and funds were granted by a private charity foundation on the basis of an application to which local stakeholders had been signatories but not significant contributors. The conceptual framework (but not concrete activities or interventions) was thus defined before local stakeholders and beneficiaries became involved. Although this is often the case in large externally funded intervention projects, it nevertheless represents a top-down approach in the early planning stages, which challenges the principles of participation and inclusiveness of the supersetting approach. Even if it may be argued that it is reasonable to secure funds before involving (and leaving expectations in) local stakeholders it is tempting to speculate that initial resistance to the initiative among local stakeholders and the need for massive efforts to motivate them could have been avoided had they been involved in the most early stages of conceptualisation, design and fundraising of the initiative. In “Health and Local Community” it took about one year to generate a proper foundation of mutual trust and respect upon which all partners (i.e. researchers and local stakeholders) could move together in pursuit of a common good, namely a healthier local community. Instead of acting for change in the local communities, much of this trust-building year was spent on talking, listening, arguing, discussing, socialising and being visible to each other while addressing the ambitions, values, principles and approaches of “Health and Local Community”. As an alternative to this post-funding “selling” of the initiative, local stakeholders could have engaged in a joint intervention mapping [49,50] or concept mapping [51] process to identify needs and interests at a pre-funding stage. This would have informed the funding application, made the initiative more locally relevant and secured early local ownership, and thus freed subsequent “selling-time” for other more action-oriented activities.

The discussion about *timing* of involvement of local stakeholders deserves to be expanded to a discussion about *strategy* and *process* of their involvement. “Health and Local Community” opted for a top-down based strategy of involvement whereby dialogue with leaders and managers in relevant health, prevention, education and child care departments of the public administration were organised prior to dialogue with education, pedagogic and health staff of their respective institutions. This served to acknowledge the public administrations’ “ownership” of public institutions while providing the initiative with a formalised authorization to interact and work with professional staff in the involved institutions. The intention was to reach agreement with local authorities about the overall framework within which the initiative should operate and thereby to create a basis for a more sustainable organisational integration of interventions. The bottom-up approach whereby problems, visions and actions would be defined by professionals, children and their parents within the local communities, was meant to unfold within this framework. Interestingly, this approach was criticized by local professionals; the initiative was seen as being imposed by higher authorities with little sensitivity to institutional resource deficiencies, time constraints and planning cycles. The professionals of the involved partner institutions considered themselves to be victims of yet another political agenda with no rights to decline rather than winners of a possibility to bring new inspiration to the their professional toolboxes. It cannot be excluded that “Health and Local Community” would have benefitted from a fine tuning of the top-down versus bottom-up balance, e.g. by involving professionals at community level earlier in the planning processes and thus reducing the influence of decision-makers (politicians and senior officers) at local government level. Although there is probably no “one-size fits all” solution to the most effective balance between top-down and bottom-up strategies in health promotion initiatives it is interesting to note that the main argument of a World Bank review of almost 500 studies on participation in community development programs around the world, is that “participatory development is most effective when it works within a “sandwich” formed by support from an effective central state and bottom-up civic action” [36]. Interestingly, the review also observes that the most successful programs tend to be implemented by local governments that have some discretion and are downwardly accountable. Devolving the management of public programs to NGOs appears to work less well. Dooris & Heritage [39] notes that the European Healthy Cities movement has demonstrated its ability to bridge the gulf between “top-down” and “bottom-up” and make an important contribution to health, well-being and sustainable development. In concordance with these observations, Dooris [31] has proposed a model for setting-based initiatives highlighting a need to balance long-term development with high-visibility project work, and top-down commitment with bottom-up engagement.

“Health and Local Community” represents an example of a supersetting initiative with a variety of interesting features: It is broadly integrated into the local community and its human and material resources; it involves ordinary citizens, mainly children and their parents; it is developed and implemented with broad local ownership and engagement; it includes wellbeing and life quality in its health perspective; it combines pedagogic, social and structural interventions; it includes multiple actions targeting the same overall goal; it operates through several sectors and settings; and it is research and knowledge-based. However, as a complex initiative it has experienced many challenges, some of which are described above. Israel et al. [52] did a comprehensive review of research-based health promotion initiatives and identified three main categories of challenges, namely 1) partnership-related issues, 2) methodological issues and 3) broader social, political, economic, institutional and cultural issues. We note that most, if not all, challenges described in this paper for “Health and Local Community” are included under these categories and thus experienced in several other community-based health promotion initiatives. This includes issues related to trust and respect; distribution of power and control; differences in perspective, priorities, assumptions and values; differences in emphases on task and process; inclusion/exclusion criteria; expectations/demands of funding institutions; political and social dynamics within the community; deterrents to institutional, community, and social change; proving intervention success; seeking balance between research and action; timing and time demands; and funding. Israel et al. also reviewed solutions adopted to meet these challenges and argues for the use of the wealth of experience about challenges and facilitating factors of health promotion initiatives to provide inspiration in the design of new initiatives.

We acknowledge the diversity of partnership-related, methodological and broader contextual challenges of research-based health promotion initiatives as described by Israel et al. [52]. We also see opportunities for addressing these challenges based on the wealth of relevant experience described in the literature. We believe that the supersetting approach and its associated values and principles contribute new inspiration for future health promotion initiatives and recommend researchers and development partners to carefully consider, from the very early stages of conceptualization, how to secure integration of interventions, broad participation of stakeholders, empowerment of citizens and communities, understanding and responding to context, and generation and use of research-based knowledge to revisit interventions. We believe that the supersetting-approach represents a useful way of thinking about how to organise and implement complex interventions for sustainable impact in community health promotion. With this paper we thus hope to promote reflection on strategies and actions for conceptualising community development initiatives, for understanding the operational obstacles that are likely to occur, and for planning which values and principles to prioritise, how to approach emerging obstacles, whom to involve as stakeholders, how to organise themselves, and how to apply scientific methods to support development and evaluation efforts. Upcoming scientific papers based on experiences from the “Health and Local Community” initiative will more elaborately address how we mobilized specific stakeholders and organized specific activities within and across the diversity of settings at community level.

## Conclusion

The supersetting approach is a relevant and useful conceptual framework for developing intervention-based initiatives for sustainable impact in community health promotion. It strives to attain synergistic effects from activities that are carried out in multiple settings either simultaneously or phased but always in a coordinated manner. The supersetting approach is based on holistic, ecological and whole-systems thinking, and stipulates important principles and values of integration, participation, empowerment, context and knowledge-based development. The Ottawa Charter states that “*The prerequisites and prospects for health cannot be ensured by the health sector alone. Health promotion demands coordinated action by all concerned” –* In recognition of this notion, the supersetting approach argues for optimised effectiveness of health promotion action by integrated efforts through long-lasting partnerships involving a diverse range of sectors, organisations, institutions, private enterprises, media, and, last but not least, ordinary people. Health governance in the 21st century is challenged to link three big debates to the health agenda, namely wellbeing, sustainability and social investment. We believe that the supersetting approach is conceptually well-positioned to become part of this new agenda and will give credit to the important values and principles first raised in the PHC and health promotion debates around 30 years ago.

## Abbreviations

BMI: Body mass index
IMF: International monetary fund
MDG: Millennium development goals
NCD: Non-communicable disease
PHC: Primary health care
UN: United Nations
WHO: World Health Organization
